# A Point-of-Care Digital Workflow for 3D Printed Passive Presurgical Orthopedic Plates in Cleft Care

**DOI:** 10.3390/children9081261

**Published:** 2022-08-20

**Authors:** Parichehr Zarean, Paridokht Zarean, Florian M. Thieringer, Andreas A. Mueller, Sabine Kressmann, Martin Erismann, Neha Sharma, Benito K. Benitez

**Affiliations:** 1 Oral and Craniomaxillofacial Surgery, University Hospital Basel, University of Basel, Spitalstrasse 21, 4031 Basel, Switzerland; 2 Medical Additive Manufacturing Research Group (Swiss MAM), Department of Biomedical Engineering, University of Basel, Gewerbestrasse 14, 4123 Allschwil, Switzerland; 3 Facial and Cranial Anomalies Research Group, Department of Clinical Research and Department of Biomedical Engineering, University of Basel, Spitalstrasse 12, 4031 Basel, Switzerland

**Keywords:** cleft lip, cleft palate, 3-Dimensional printing, computer-aided design, presurgical orthopedics, intraoral scanning

## Abstract

Cleft lip and palate are one of the most common congenital craniofacial malformations. As an initial treatment, presurgical orthopedics is considered standard treatment at many cleft centers. Digital impressions are becoming feasible in cleft care. Computer-aided design (CAD) and three-dimensional (3D) printing are manufacturing standards in dentistry. The assimilation of these technologies has the potential to alter the traditional workflow for the fabrication of customized presurgical orthopedic plates. We present a digital workflow comprising three steps: 3D digital image acquisition with an intraoral scanner, open-source CAD modeling, and point-of-care 3D printing for the fabrication of personalized passive presurgical plates for newborns with cleft lip and palate. The digital workflow resulted in patient-related benefits, such as no risk of airway obstruction with quicker data acquisition (range 1–2.5 min). Throughput time was higher in the digital workflow 260–350 min compared to 135 min in the conventional workflow. The manual and personal intervention time was reduced from 135 min to 60 min. We show a clinically useful digital workflow for presurgical plates in cleft treatment. Once care providers overcome procurement costs, digital impressions, and point-of-care 3D printing will simplify these workflows and have the potential to become standard for cleft care.

## 1. Introduction

Presurgical orthopedics has a long history of development and application in patients with cleft lip and palate [1,2,3,4]. More than half a century after McNeil’s introduction, presurgical orthopedic therapy aims to narrow the cleft, align the alveolar cleft segments, and improve nasal symmetry to allow for surgical repair under minimum tension and tissue shift [1,5,6]. Various appliances exist with pin retained active plates on one end of the spectrum and passive plates on the other [2,3,4,5,7,8].

Different presurgical concepts share the need for an oral maxillary impression to manufacture presurgical plates. As conventional impressions are a risk for the infant’s airway [9,10], digital impression techniques, not having the risk of impression material aspiration or ingestion, provide a safer alternative. Digital impressions have become feasible in cleft care also for newborns and have been introduced in different centers [11,12,13]. Furthermore, digitization and three-dimensional (3D) printing increased the use of computer-aided design and manufacturing (CAD-CAM) in cleft care [14,15,16]. This development allows to produce dental appliances, providing new realms of digitization. The availability of 3D printing in hospitals and the use of biocompatible materials enables treating patients without delays [17].

The assimilation of these technologies contributes to patient-specific digital data in a less invasive manner and has the potential to alter the traditional workflow for the fabrication of customized presurgical orthopedic plates. Our aim was to provide a clinician-friendly, easy to implement digital workflow at the point-of-care in cleft care, utilizing the current capabilities of digital technologies. We developed a clinically usable 3D design and 3D printing approach for the fabrication of personalized passive presurgical orthopedic plates for newborns with cleft lip and palate.

## 2. Materials and Methods

This section introduces an interactive digital design approach for personalized passive presurgical orthopedic plates for patients with cleft lip and palate. The setup of the digital workflow comprised three phases: 3D digital image data acquisition, computer-aided design (CAD) modeling, and 3D printing.

### 2.1. Three-Dimensional (3D) Digital Image Data Acquisition

Before digital image acquisition, we calibrated an intraoral scanner (Medit i500, Medit Corp, Seoul, Korea) according to the manufacturer’s instructions. The scanner works with 3D-in-motion imaging video technology, allowing data capturing while the infant is awake. The scanner’s acquisition software automatically calculated the scan duration and registered the cleft deformity. Systematic capturing of the entire cleft malformation with the depth of the vestibular fold, the labial and buccal frenulum, as well as the extension of the distal area and tuberosity was essential for delineating the flange extension of the subsequent passive plate. Lastly, using the scanner’s integrated post-processing tools, a 3D image of the entire cleft morphology was created and exported in a standard tessellation language (STL) file format.

### 2.2. Computer-Aided Design (CAD) Modeling

In this section, we implemented a sequential design phase for the cleft anatomical model and for the passive presurgical orthopedic plate. To create a 3D anatomical cleft model, we imported the STL file into an open-source CAD software (Meshmixer v. 3.5.474, Autodesk Inc., San Rafael, CA, USA). We cleaned the scan data using the software’s *sculpt tool* to remove any unnecessary image artifacts and overlapping triangles. Using wireframe functionality, we checked the image data for equidistant mesh tessellations and remeshed, if needed. Subsequently, we hollowed the scan data using the *edit tool* with a 2 mm offset distance, creating the model’s outer wall thickness. This allowed to reduce the overall printing time and material consumption. Finally, we exported the anatomical cleft model as an STL file for further printing processes.

Experience in treating cleft lip and palate, as well as presurgical orthopedics is required to digitally select and mark the alveolar ridge seating surface. Care must be taken to ensure that the seating surface of the passive plate does not cause pressure marks, especially on the labial or buccal frenulum, the vestibular fornix, and the vomer. Therefore, to design a passive presurgical plate, we blocked all undercut regions with the *sculpt tool* to fill the palatal and alveolar cleft to mimic a normal palatal shape. Next, we marked the region of interest for the plate extent. After smoothing the boundaries, we separated the plate and extracted it with an offset value of 0.1 mm, keeping the connected functionality unchecked. The region between the plate and model was deleted by inverting the selected area. The plate’s thickness was then determined. We used the *offset tool* to extrude the plate’s surface to a thickness of 1.8 mm with the surface-connected functionality turned on. The plate’s borders and outer surface were optimized by removing sharp edges before exporting the plate’s STL file.

### 2.3. 3D Printing of Anatoical Cleft Model and Passive Presurgical Orthopedic Plate

We fabricated the anatomical cleft model and the passive presurgical orthopedic plate using Stereolithography (SLA) 3D printer (Formlabs 3B, Formlabs Inc., Somerville, MA, USA). We printed the cleft anatomical model in a non-biocompatible photopolymer resin material (Model resin, Formlabs Inc., Somerville, MA, USA), while we fabricated the passive plate in a biocompatible photopolymer resin material (BioMed Clear, Formlabs Inc., Somerville, MA, USA). The latter is a hard, medical-grade biocompatible material certified as U.S. Pharmacopeia Class VI for long-term skin or mucosal membrane contact and compatible with conventional sterilization methods [18]. We imported the model and passive plate STL files into the printer’s slicing software (Preform v. 3.19.0, Formlabs Inc., Somerville, MA, USA), and the representative material’s printing parameters were defined. The anatomical model and passive plate were printed at a layer thickness of 100 μm. We manually defined the model’s orientation with the intaglio surface facing away from the build platform. Similarly, for the passive plate, care was taken during print orientation to place support structures away from the later palatal contact area.

After completion of the printing process, we visually inspected the anatomical cleft model and passive plate for printing defects or errors. Postprocessing procedures were followed to remove the uncured resin from the printed part surface. The model and plates were transferred into a postprocessing unit for washing and curing procedures. For washing, printed parts were inserted into the Form Wash device (Formlabs Inc., Somerville, MA, USA) filled with Isopropyl alcohol concentration of 90% or higher for 20 min, followed by air drying for at least 30 min. Consequently, to attain good mechanical properties of the biocompatible resin material, the following post-curing procedure for 60 min at 60 °C (405 nm, 100 W) was carried out using Form Cure (Formlabs Inc., Somerville, MA, USA) as per the manufacturer’s instructions [19].

Finally, we removed the support structures manually by finish kit (Formlabs Inc., Somerville, MA, USA) including flush cutters and tweezers and delivered the anatomical model with the plate to the dental technician for final polishing.

## 3. Results

**Figure 1:**
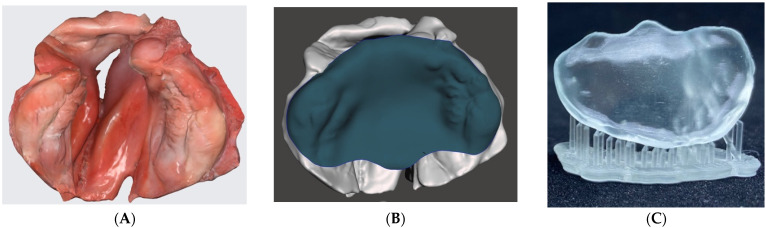
Overview representation of the digital workflow for the fabrication of a passive presurgical orthopedic plate in a unilateral cleft lip and palate exemplary case. (**A**) Intraoral three-dimensional (3D) digital image acquisition; (**B**) computer-aided plate design (CAD) modeling of presurgical orthopedic plate; (**C**) three-dimensional (3D) printed presurgical orthopedic plate.

Figure 1 shows the results of the described digital workflow from digital image data acquisition with an intraoral scanner, 3D designing, and 3D printing for the fabrication of a personalized passive presurgical orthopedic plate. A sequential and detailed step-by-step illustration of the digital workflow is accessible under Open Science Framework (Supplements S1–S4).

### 3.1. Three-Dimensional (3D) Digital Image Data Acquisition

**Figure 2:**
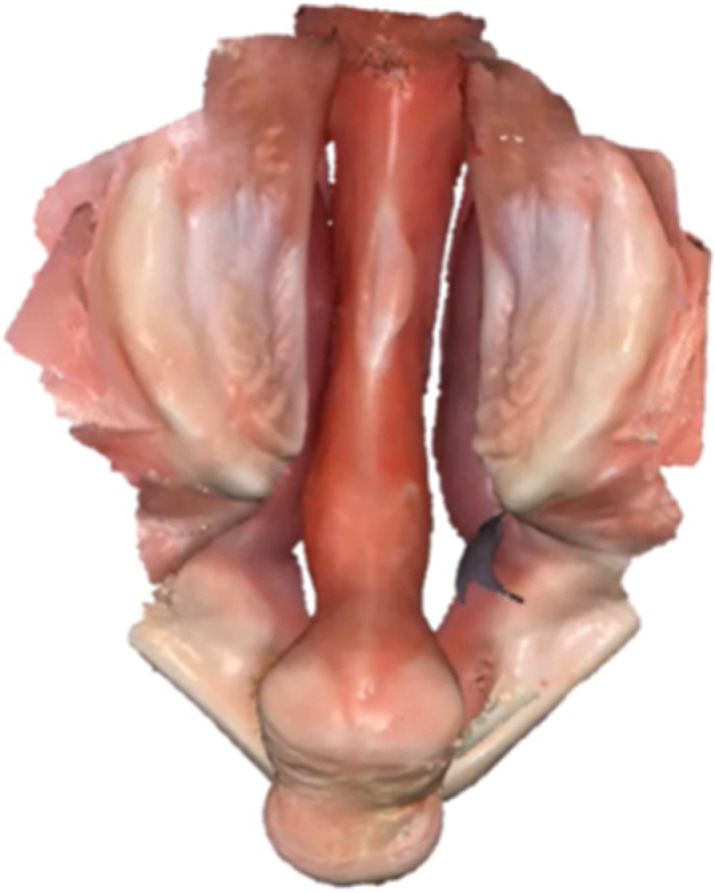
Digital impression example in an infant with bilateral cleft lip and palate. The Video S1 shows data acquisition with the Medit i500 intraoral scanner and is available under Open Science Framework.

The total time required for an intraoral scan was dependent on the complexity of the cleft anatomy and surface area to capture. Image data acquisition in awake neonates and infants could last less than 1 min for patients with cleft palate only, and up to 2.5 min when capturing the anatomy of bilateral cleft lip and palate [13]. Figure 2 and Video S1 shows an example of a digital impression taken with the Medit i500 intraoral scanner in a patient with bilateral cleft lip and palate, with the corresponding time for scanning (2 min 25 s), indicated in the upper right corner (Open Science Framework).

### 3.2. Computer-Aided Design (CAD) Modeling

**Figure 3:**
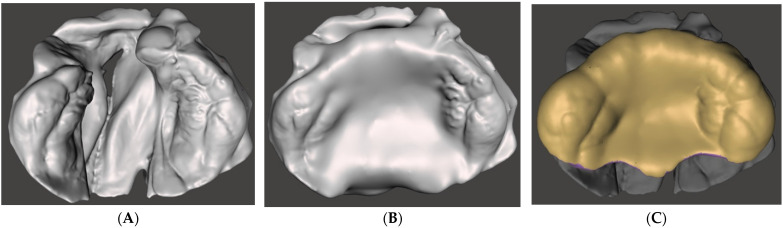
Unilateral cleft lip and palate model (**A**) as imported; (**B**) with blocked cleft area to mimic a normal palatal shape; (**C**) final passive presurgical orthopedic plate ready for 3D printing.

**Figure 4:**
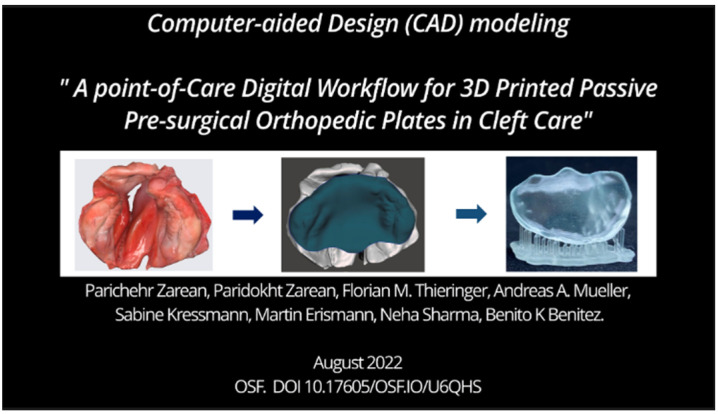
Condensed video tutorial for computer-aided design (CAD) modeling with open-source CAD software Meshmixer for a passive presurgical orthopedic plate, available as Video S2 under Open Science Framework.

Figure 3 illustrates an overview of the 3D CAD modeling steps for the passive presurgical orthopedic plate. The time required for the computational design phase was around 15–20 min. Supplement S2 shows the design steps for the cleft anatomical model and Supplement S3 shows sequential and detailed design steps for the passive presurgical orthopedic plate. The corresponding STL files of the model and plate and a condensed video tutorial for CAD modeling (Figure 4 and Video S2) are accessible under Open Science Framework.

### 3.3. 3D Printing of Anatomical Cleft Model and Passive Presurgical Orthopedic Plate

**Figure 5:**
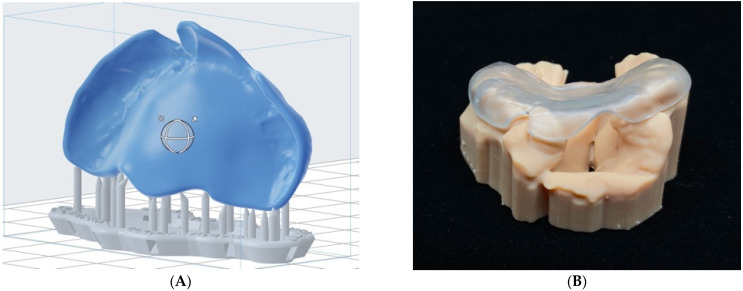
(**A**) Manual-orientation of the presurgical plate and auto-generation of the support structure. (**B**) Digitally designed and 3D printed passive presurgical plate with corresponding anatomical cleft model.

For the fabrication phase, the total printing time of a plate and anatomical model was around 90–180 min in the exemplary case of unilateral cleft lip and palate. The total printing time depended on the size of the anatomical model, thus on the child’s age and the depth of the cleft, i.e., the height of the anatomical model. An additional 60 min of automated post-processing for the representative resin materials were needed. Figure 5A shows the orientation of the presurgical plate (blue color) on the 3D printer’s build platform to generate minimum support structures (grey color), thereby minimizing the final manual finishing steps to 10 min. Figure 5B illustrates a 3D printed anatomical model in unilateral cleft lip and palate and the appropriate passive fit of the presurgical plate. Supplement S4 shows the step-by-step 3D printing process accessible under Open Science Framework.

### 3.4. The Clinical Transition from Conventional to a Digital Workflow for Presurgical Treatment

**Figure 6:**
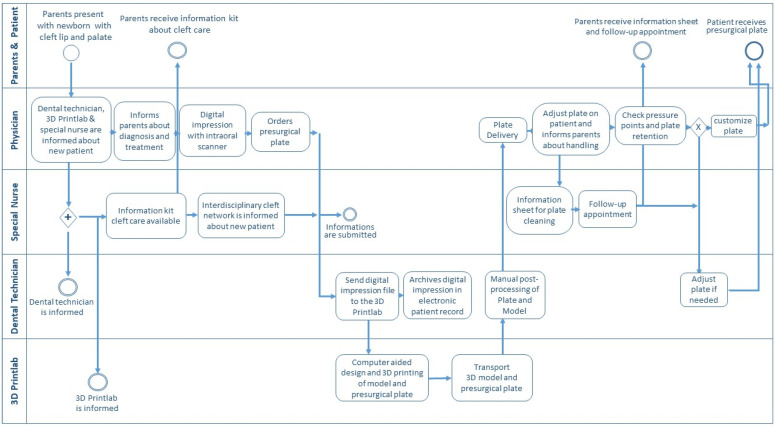
A cross-functional clinical process-oriented flowchart of the digital workflow for presurgical treatment for patients with cleft lip and palate.

Treatment of patients with cleft lip and palate encompasses a multi-disciplinary team. The transition from conventional to digital workflow required a cross-functional process alignment. Therefore, a schematic representation of the digital workflow addressing care and medical team involved in presurgical orthopedic treatment is presented in a process-oriented flowchart (Figure 6). The parents, the treating physician, the cleft special nurse, the dental technician, and the point-of-care 3D printing team were involved. The process describes the roles and tasks, starting with parents presenting with their newborn with cleft lip and palate at the interdisciplinary cleft center. The roles and tasks of different cleft team members involved were clarified with the four key responsibilities (responsible, accountable, consulted, and informed).

**Figure 7:**
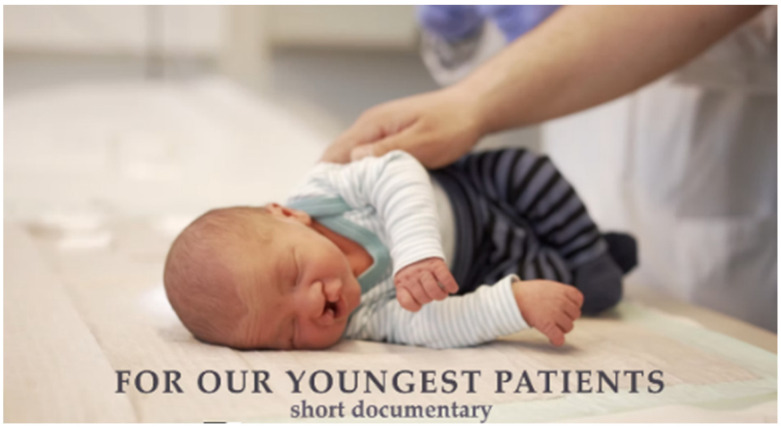
A short documentary for parents about the rationale and the process of the digital workflow for presurgical treatment in cleft lip and palate (accessible on Vimeo).

Furthermore, based on our center experience, a comparison of conventional impression taking and presurgical plate manufacturing with the current digital workflow is illustrated in Table 1. It was noticed that the digital workflow results in more patient-related benefits, such as a lack of risk of airway obstruction with a quicker data acquisition for the patient and reduced stress for the clinicians as well as parents by having parents/caregivers next by the clinician during an intraoral scan. The digital model was used directly for parent education and to demonstrate the progress of treatment. Additionally, a reduced amount of laborious manual time for the technician was noticed. The digital workflow provided direct digital data storage and archiving in the electronic patient chart, compatible with the obligation to store medical data and beneficial for further outcome evaluations. Analyzation of throughput time showed 260–350 min for the digital workflow compared to 135 min in the conventional workflow. However, the digital workflow resulted in less manual and personnel intervention with the hands-on time reduced from 135 min to 60 min. A documentary for parents about the rationale and the process of the digital workflow for presurgical orthopedic treatment based on a patient story is provided on Vimeo (Figure 7 and Video S3).

## 4. Discussion

Digital intraoral scanning is on the verge of replacing traditional impression techniques and is currently used in various areas of dentistry with promising results [20]. Digital impressions do not pose risks to the airway, require fewer personal resources, and allow direct 3D data export. Consequently, we switched from conventional to digital impressions in our daily clinical routine [13]. With rapid advances in digital technology in dentistry and surgery, new digital workflows must be implemented in the clinical routine. We established a complete digital workflow for point-of-care design and 3D printing of presurgical passive orthopedic plates for cleft care.

Presurgical orthopedic plates are used in cleft lip and palate for years, and different concepts from active to passive molding exist [1,12]. Despite controversies on the use of presurgical orthopedics, most American Cleft Palate-Craniofacial Association (ACPA) approved and registered international teams provide presurgical infant orthopedics [21]. At our center, we use passive presurgical treatment to separate the oral and nasal cavity, keep the tongue out of the cleft for normalized intraoral forces, and consequently observe a passive reduction of cleft size before cleft palate repair [22].

**Figure 8:**
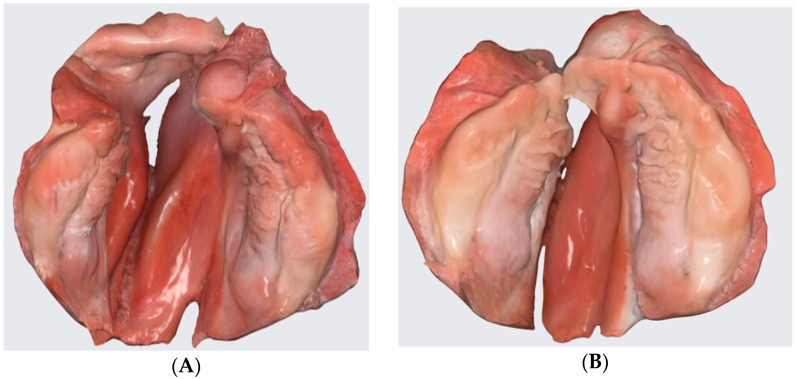
Intraoral scans of a unilateral cleft lip and palate (**A**) at birth and (**B**) after two months of treatment with a passive presurgical plate.

At the center of the authors, the costs for presurgical treatment are covered by insurance. Presurgical orthopedics is started at our center from the first days after birth. In passive plate therapy, the contact of the plate is limited to the alveolar ridges with a spacing between the alveolar cleft and the palatal cleft. As a result, the contact area is smaller compared to other concepts, e.g., Hotz plate [2] and the passive plate is used with a small amount of dental adhesive cream (Kukident, Permadental GmbH, Emmerich am Rhein, Germany) in the area of the alveolar ridges. Parents are instructed to clean and inspect the mouth and disinfect the plate once a day (Ocenisept, Schülke and Mayer GmbH, Norderstedt, Germany). Otherwise, the plate is worn 24 h/day. Clinical follow-up is planned after seven days for potential pressure sores. Apart from that, no additional visits for plate adaptations and grinding procedures are required. Depending on growth of the infant, renewal of the plate is required. The first passive plate is used for up to three to four months in unilateral cleft lip and palate. This is due to the continuous reduction of the cleft by approximation of the greater and lesser segment observed under presurgical treatment [22], compensating for the growth of the segments. The second plate is worn until combined cleft nose, lip, and palate repair in one single surgical intervention is performed after eight months of age [6,23]. Early secondary alveolar bone grafting is then performed around five to six years of age before the permanent teeth adjacent to the cleft erupt. Figure 8 shows a complete unilateral cleft lip and palate at birth and two months follow-up with passive presurgical therapy.

Traditionally plates are made from acrylic resin materials or vacuum-adapted low-density polyethylene material (ethylene vinyl acetate) [24]. This fabrication process is manually laborious and time-consuming. Advancements in digital technologies allow for more efficient workflows, producing customized dental appliances previously based on laborious manual handcrafting procedures [24]. The development of CAD and 3D printing allow to produce dental appliances at the point-of-care [25]. It enabled us to manufacture patients-specific plates at the earliest time possible.

Other authors have used commercially available CAD software solutions in their digital workflow [11,12,16]. Since there are no straightforward, affordable, and accessible software solutions, the difficulty of developing a practical and workable virtual workflow persists, which might hinder implementation and knowledge transfer to other cleft centers, especially with limited resources [12]. In contrast, our workflow utilizes an open-source CAD software with no additional costs and offers the possibility to design other types of presurgical orthopedics appliances. 3D printing enables effective production only after the plate has been digitally generated. By providing an intuitive software solution and the option to outsource the production process, it may be able to expand the accessibility of this application.

Photopolymer resin materials are used to 3D print dental appliances, surgical and cutting guides made of biocompatible materials [17]. With the accessibility of in-house 3D printing, creating and fabricating presurgical orthopedics plates has been transferred from the external service providers to the hands of clinicians. Clinicians can now choose from various 3D printers and materials with proven biocompatibility thanks to ongoing advances in 3D printing technology [17]. Furthermore, manufacturers of 3D printers are refining their machines and making their systems accessible to additional third-party materials produced by other manufacturers. The ability to select from various affordable and certified biocompatible resin materials offers clinicians a desirable alternative. The materials cost estimated in our exemplary case was 2 Euro for the presurgical plate and 3.5 Euro for the anatomical model. These piece costs were in the range of other groups using 3D printing in cleft care [26].

### 4.1. Clinical Relevance

Integration of digital technologies for presurgical plate production in the treatment of cleft patients represents a unique and promising field that is still in its early stages. This work is the first step in a project to automate, simplify, and reduce manpower in a promising passive plate therapy for presurgical cleft lip and palate treatment. Changes from conventional to a digital workflow for presurgical treatment provide a variety of benefits. To summarize, the switch to digital impression taking and 3D design, and printing required adaptation to a higher turnaround time, but brought more benefits such as reduced risk and resources for impression taking, especially relevant in patients with Robin Sequence, e.g., for treatment of airway obstruction with a Preepiglotic Baton Plate [27,28], as well as reduced stress for clinicians and parents by having parents/caregivers next by the clinician. Furthermore, direct digital data storage, digital measurements, and outcome evaluation are possible. Digital impression taking was clinically preferred as it offered a more detailed representation of cleft morphology without artifacts that we had previously seen when applying pressure with the impression material. This clinical superiority of digital impressions, however, no longer justified a study comparing the accuracy of conventional and digital impressions in our vulnerable patient population, as there was no true equipoise. The higher precision was clinically evident in fewer pressure sores and significantly less need for plate adjustments on the patient. With passive plates, we have no grinding and fewer patient visits with the same plate for three to four months which could be considered an advantage of this workflow in low resources and high-volume centers.

### 4.2. Limitations

The procurement cost of intraoral scanners and 3D printers, as well as complying with local medical device regulations, are among the limitations of the latest technology. However, outsourcing 3D printing to a dental laboratory instead of the point-of-care is a hybrid solution. However, this would not only increase the costs due to transport but also the lead times. A further limitation is the required user input and knowledge of the CAD software. In a short time, however, machine learning will enable automated plate designs with minimal to no user input [29].

The reported piece costs do not reflect the total costs, including one-time procurement costs (e.g., computer, intraoral scanner, 3D printer, postprocessing units and tools), recurring costs (consumables, resin material, tank, build platform, isopropyl alcohol), and overhead costs. Further comprehensive cost analysis is needed, considering the throughput time of digital production workflows. The workflow is limited to passive presurgical orthopedic plates. With adaptations, it could also be used for other forms of presurgical treatments. In contrast, nasoalveolar molding has been criticized for the high treatment burden, visits, and thus healthcare costs [30]. It was shown that these costs could be reduced with passive presurgical treatment [31]. This could also counteract early cleft lip surgery in the first weeks of life, which has been propagated to reduce the burden of health care costs [32].

## 5. Conclusions

The presented workflow with open-source digital design software and detailed instructions accessible on Open Science Framework will enable clinics to implement digitalization in presurgical cleft care. As intraoral scanners, digital design software, 3D printers and materials evolve, clinicians will have more opportunities to use them in their clinical routine. Aligned workflows requiring minimal interfaces from digital scanning to digital design and 3D printing are necessary to keep the process streamlined and implemented in clinics. Once healthcare providers overcome procurement costs, digital impressions, and point-of-care 3D printing will simplify these workflows and have the potential to become standard for cleft care. With the rapid pace of digital transformation in healthcare and the reduced patient risk of digital impressions and resources required, demand from parents, caregivers, and healthcare organizations is also conceivable.

## Figures and Tables

**Table 1:** Comparison of conventional impression taking and presurgical plate manufacturing with the current digital workflow.

	Conventional Workflow	Digital Workflow
**Impression**		
Available readiness of anesthesiology, neonatology team (some centers take an impression in the operating theater)	Yes	No
Involvement and presence of parents/caregivers; Parent education, demonstration of treatment progress	No	Yes
Known risk for the airway	Yes	No
Added stress for clinician	Yes	No
Time for setup material and impression taking	30 min	15 min
The time for impression taking by the physician (conventional vs digital)	2–5 min	1–2.5 min
Time to obtain a physical plaster cast model	35 min (technician)	N/A
Detail accuracy of the model	Low	High
Digitalization of 3D model for archiving	10 min (scanning required)	automatic
**Presurgical Plate Fabrication**		
Computer-aided design modeling	N/A	35 min
3D printing time	N/A	90–180min *
Postprocessing printed parts	N/A	110min *
Hands-on plate manufacturing on the plaster cast model	45 min	N/A
Final preparation and polishing of presurgical plate	15 min	10 min

* In these times no personnel intervention is needed. The total printing time is variable depending on the size of the anatomical model.

## Data Availability

All data supporting reported data can be found under Open Science Framework and Vimeo.

## Supplementary Materials

The following supporting information is accessible under Open Science Framework (https://osf.io/u6qhs/) and Vimeo (https://vimeo.com/697305051). Supplement S1–S4: Step-by-step illustration of the digital workflow; Video S1: Digital impression example in an infant with bilateral cleft lip and palate; Video S2: Condensed video tutorial for computer-aided design (CAD) modeling with open-source CAD software Meshmixer for a passive presurgical orthopedic plate; Video S3: A short documentary for parents about the rationale and the process of the digital workflow for presurgical treatment in cleft lip and palate.
